# Occurrence of Human Norovirus and Hepatitis A Virus in Domestic Agricultural Waters

**DOI:** 10.14252/foodsafetyfscj.D-25-00006

**Published:** 2026-06-26

**Authors:** Christine Yu, Qianru Yang, Michael Kulka, Erin Lipp, Michael Kauffman

**Affiliations:** 1 Office of Applied Microbiology and Technology, Office of Laboratory Operations and Applied Science, Human Foods Program, US Food and Drug Administration, Laurel, Maryland 20708, USA; 2 Department of Environmental Health Science, University of Georgia, Athens, Georgia 30602, USA; 3 Center for Food Animal Health, The Ohio State University, Wooster, Ohio 44691, USA

**Keywords:** agricultural water, dead-end ultrafiltration, hepatitis A virus, human norovirus, modified Moore swab

## Abstract

Assessing the prevalence of foodborne pathogens in the environment is an important step toward mitigating those risks. In this comprehensive two-year study, we assessed the occurrence of human norovirus (NoV) and hepatitis A virus (HAV) in agricultural water used for crop irrigation to better understand potential sources of contamination, which can contribute to outbreaks caused by these viruses. For the first series of experiments, samples of surface and ground water were collected monthly during the growing seasons from 36 sites, representing 21 farms in two regions in the US, namely Georgia (GA) and Ohio (OH). Two primary concentration methods were employed to capture naturally occurring viruses from 10L of water: either dead-end hollow fiber ultrafiltration (DEUF) or modified Moore swab (MMS). For secondary virus concentration, DEUF was coupled with polyethylene glycol (PEG) precipitation, while MMS was paired with ultracentrifugation. The detection rate using RT-qPCR was 8.4% for NoV genogroup II (NoV-GII) and 9.4% for HAV, found across 24 sampling sites on 15 farms. We then directly compared the performance of DEUF and MMS for virus recovery by concentrating 10 L of agricultural water spiked with NoV with each method. The DEUF consistently provided both higher detection rates and lower Ct values across various levels of virus spiking (p < 0.05 at the seeding level of 5.0E+05 genome copies/10L). There were no significant differences in virus recovery between the two secondary concentration methods (PEG or ultracentrifugation). Improving methods for obtaining surveillance information will provide greater insights into the prevalence of human enteric viruses in irrigation water and support the development of viral pathogen mitigation strategies in the prevention of foodborne outbreaks.

## Introduction

Human enteric viruses such as norovirus (NoV) and hepatis A virus (HAV) are leading causes of acute viral gastroenteritis. As these viruses are shed in the feces of infected individuals and transmitted via the fecal-oral route, exposures can occur through ingestion of contaminated water or foods (such as fresh produce) that were irrigated and/or processed post-harvest using water contaminated with human waste^1^^)^. It is also possible for plants to internalize these viruses by root uptake of polluted agricultural water^2^^,^^3^^,^^4^^,^^5^^)^. Foodborne outbreaks associated with these viruses have been increasing in recent years, concomitant with the increasing consumption of fresh fruits and vegetables that have been linked to exposure of these foods to virus-contaminated irrigation and processing water^6^^,^^7^^)^.

Sewage is a major source of contamination of the agricultural environment with human enteric viruses. Agricultural fields, surface water, ground water, and reclaimed water have been documented to be contaminated with human waste with the potential to transmit diseases^8^^,^^9^^,^^10^^)^. In one study by Tian et al., NoV was detected in 25.6% of water samples collected from an agricultural region in the central California coast^11^^)^. A Canadian committee reviewed 22 studies in which enteric viruses had been detected in surface waters and groundwaters of North America; the occurrence of enteric viruses (adenovirus, norovirus, enterovirus) ranged from 6.8% to 82% for surface water and 0% to 46% for groundwater^12^^)^. Studies have also demonstrated that norovirus levels in raw wastewater exhibit significant geographical or seasonal variations (higher in the winter than in the summer)^13^^,^^14^^)^.

Given the importance of human enteric viruses as etiologic agents of foodborne and waterborne diseases, there have been continuing efforts to assess the prevalence of these viruses in the agricultural environment for outbreak investigations, risk assessments, and development of prevention strategies. However, such assessments are challenging, as infectious doses for both HAV and NoV are quite low, estimated in the range of 10 – 100 viral particles, which may be due in part to their capsid stability^15^^,^^16^^)^. This suggests that even low amounts of virus contamination could cause illnesses, even though such low levels in agricultural water sources might be below the limits of detection. To compensate for the low concentrations of viruses in natural water, two multi-step concentration methods for large-volume sampling have been used to enhance the effectiveness of detection assays. For the initial concentration, dead-end hollow fiber ultrafiltration (DEUF) provides filtration by size exclusion while modified Moore swabs (MMS) function by adsorption filtration. Both these approaches have been proven effective for the recovery of various microbes (viruses, bacteria, parasites) from large volumes of water^11^^,^^17^^,^^18^^,^^19^^,^^20^^,^^21^^)^. For secondary concentration, polyethylene glycol (PEG) precipitation and ultracentrifugation are well-established methods for achieving smaller volumes and concentrating viruses from aqueous samples prior to nucleic acid extraction and downstream molecular detection methods, such as RT-qPCR and sequencing. However, PEG can be applied to a wide range of sample volumes (from mL to L); in contrast, the sample volume for ultracentrifugation is often constrained by the capacity of the ultracentrifuge tubes (mL).

These multi-step collection and concentration methods were applied in a two-year surveillance study to better understand the prevalence of NoV and HAV in the agricultural water systems of farms in two regions in the US, Ohio (OH) and Georgia (GA). The study surveyed the traditional irrigation water sources (*i.e.*, both surface and ground waters: creeks, streams, ponds, wells, and drip tapes). Detection of HAV and NoV genotypes I and II (GI and GII, respectively) was based on reverse transcription-quantitative PCR (RT-qPCR) analysis. We then compared the performance of the two primary concentration methods (DEUF and MMS) and the two secondary concentration methods (PEG precipitation and ultracentrifugation) by using artificially spiked agricultural water samples.

## Materials and Methods

### Virus Strains

For the spiking portion of our study, the human NoV strain (GenBank accession number MK301293, NoV-GII.6[P7]) was obtained from a sporadic case of acute gastroenteritis^22^^)^. The initial stock was prepared as a 10% (wt/vol) fecal suspension in phosphate-buffered saline (PBS). The homogenate was centrifuged at 13,000 × g, room temperature, for 3 min. The supernatant was aliquoted to new tubes and used as the viral stock. Viral titer of the stock was determined to be approximately 3 × 10^9^ genome copies (gc) per mL measured by RT-qPCR. These viral stocks were stored at -80°C until use.

### Sampling Locations

Farms where water samples were collected were recruited through voluntary agreements with the farmers. All farms were blinded for the purposes of this study. Thirty-six sampling sites (ponds, wells, creeks, streams, or the end of drip tapes) on twenty-one farms: 4 farms with 11 sites in GA and 17 farms with 25 sites in OH, as selected by the collaborators. The pH and oxidation-reduction potential (ORP) of water were measured in Ohio water sources. The temperatures of water during sampling were recorded in both OH and GA.

### Sample Collection and Filtration

Agricultural water samples from ponds, streams, creeks, and drip tapes were collected at approximately 1-month intervals during the growing seasons over a 2-year period (May to December in 2019 and June to September in 2020). For each sample, ten liters of water were filtered through either MMS or DEUF filters. The exception was samples of drip tape water, for which only one liter water could be collected using either filtration method (DEUF or MMS); this was due to the low collection flow rates and frequent clogging of the filters that occurred when attempting to filter larger volumes.

Modified Moore swabs were hand-made by inserting rolled-up gauze into polyvinyl chloride (PVC) cartridges described by Sbodio et al.^23^^)^. After the filtration collection, these swabs were placed into Whirl-Pak bags (Nasco, Fort Atkinson, WI), kept in an ice-packed cooler, transported overnight to the lab, and stored in a refrigerator until processing. For each DEUF filtration, a single-use Rexeed-25S filter with a molecular mass cutoff of 30 kDa (Asahi Kasei Medical Co. Ltd., Tokyo, Japan) was pre-blocked with 5% bovine calf serum by filtering 500 ml blocking solution followed by 2h incubation with shaking at 100 rpm, room temperature^17^^)^. Afterward, each pre-blocked DEUF filter was flushed with 1 L of sterile water before the selected agricultural water source for sampling was filtered according to the FDA BAM Chapter 19c^24^^)^. After sample collection, the DEUF filters were transported and stored in a similar manner to the MMS. In 2019, a total of 100 MMS samples were collected. In 2020, a total of 210 samples (129 DEUF and 81 MMS) were collected.

### RNA Recovery from MMS Samples

**Fig. 1.**
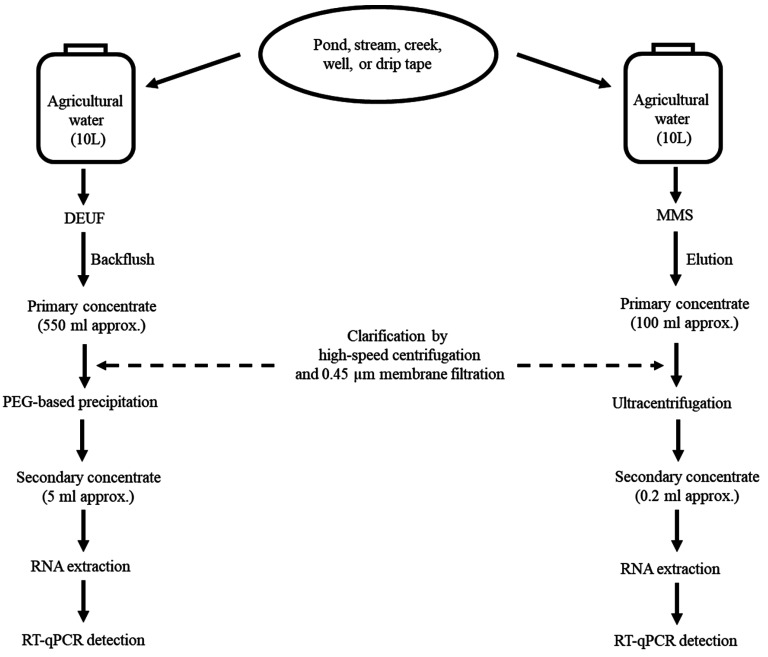
Schematic diagram of DEUF and MMS Sample Preparation and Analysis. Ten liters of agricultural water were filtered with either DEUF or MMS. For DEUF, the ultrafilter was backflushed, and the effluent was clarified followed by PEG precipitation. For MMS, the swab was eluted, and the eluate was clarified followed by ultracentrifugation. In both methods, RNA was extracted from the resuspended pellet and analyzed by RT-qPCR. DEUF, dead-end hollow fiber ultrafiltration. MMS, modified Moore swab. PEG, polyethylene glycol.

Swabs were eluted with a high pH alkaline glycine-saline buffer TGBE (0.1 M Tris-HCl, 0.05 M glycine, 1% beef extract, pH 9.2) to release viral particles (**Fig.1****.)**. Briefly, 60 mL of TGBE was added to the swab in a Whirl-Pak bag and the bag was shaken at 170 rpm for 1 hr at room temperature. The eluate was clarified by centrifugation at 10,000 x g, 20 min, 4°C to remove most of the large debris. The supernatant was passed through a 0.45 µm pore-size membrane filter (Thermo Fisher Scientific, Waltham, MA). The filtrate was transferred to ultracentrifuge tubes and centrifuged at 37k rpm for 1 hr at 4°C. The pellet was resuspended with 200 µL of PBS total for each sample and stored at -20°C until RNA isolation. The virus concentrate was lysed with 500 µL of 6M guanidinium thiocyanate (GITC) followed by RNA extraction using RNeasy mini kit (Qiagen, Germantown, MD) according to the manufacturer’s instructions, with minor modifications in which a 15-min hold time was given for each washing step^13^^)^. RNA was eluted in 100 µL of TE buffer (10 mM Tris, 0.1 mM EDTA, pH 8.0) and stored frozen at -80°C until analysis.

### RNA Recovery from DEUF Samples

To recover viruses from the DEUF filtration, the Rexeed-25S filter was backflushed using 500 mL of backflush solution (0.01% Tween 80, 0.01% sodium polyphosphate, 0.001% antifoam)^25^^)^ (**Fig. 1.)**. The backflush liquid was clarified by centrifugation at 10,000 x g, 20 min, 4°C, followed by filtration through a 0.45 µm pore-size membrane filter. A secondary concentration of viruses was performed with polyethylene glycol (PEG) precipitation at 12% PEG, 0.9M NaCl, and 1% BSA and incubated for 2 hrs at 4 °C, pH 7–7.4^26^^)^. After centrifuging at 10,000 ×g for 30 min, pelleted material was resuspended in 2 mL of PBS (Dulbecco’s modification) containing 0.01% (V/V) Tween 80 and 0.001% Antifoam Y-30 Emulsion (V/V). The virus concentrate was lysed with 5 mL of 6M GITC followed by RNA extraction using QIAGEN RNeasy Maxi Kit. RNA was eluted in 800 µL of TE buffer and further concentrated to 100 µL using an Amicon Ultra-2 centrifugal filter 100kDa MWCO device (Sigma, St. Louis, MO).

### Artificial Inoculation of Agricultural Water with NoV-GII.6[P7]

For the method comparison study, water was collected from a stream on an OH farm and stored at 4°C until use. This water was spiked with a NoV-GII.6[P7] virus-positive fecal suspension either prior to DEUF/MMS filtration or after elution to evaluate the efficacy of virus recovery, RNA extraction, and the presence of RT-qPCR inhibitors. In the pre-filtration inoculation conditions, 100 µL of serially diluted NoV-GII.6[P7] in PBS was spiked to 10 L of water followed by DEUF or MMS filtration. In the post-elution inoculation condition, 100 µL of serially diluted NoV-GII.6 was spiked into either 550 ml of DEUF backflush liquid or 60 ml of MMS eluant. Seeding levels per sample ranged between 5.0E+05 and 5.7E+01 gc. Analyses of both spiked samples and non-spiked controls were carried out in triplicate. Spiked samples were processed in the same ways as the natural samples described earlier in the surveillance study; however, an additional purification step using Zymo OneStep PCR Inhibitor Removal Kit (Zymo Research, Irvine, CA) was applied to the eluted RNA.

### RT-qPCR Detection of Viruses

RNA samples were analyzed according to the protocols in the FDA BAM Chapter 26 for the detection of HAV, NoV-GI and -GII on an ABI 7500 system (Life Technologies, Carlsbad, CA) using OneStep RT-qPCR kit (Qiagen)^27^^)^.

The protocol for HAV detection included a 50 min reverse transcription reaction at 50°C and an inactivation step of 15 min at 95°C, followed by 50 cycles of 95°C for 10 sec, 53°C for 25 sec, and 64°C for 70 sec. This HAV assay used primers and probe targeting the 5’ untranslated conserved region of the genome^28^^)^ [forward primer GAR2F: 5’-ATA GGG TAA CAG CGG CGG ATA T-3’, reverse primer GAR1R: 5’-CTC AAT GCA TCC ACT GGA TGA G-3’, probe GARP: Cy5-5’-AGA CAA AAA CCA TTC AAC GCC GGA GG-3’-IB-RQ].

For detection of NoV, the same protocol was followed, except during the extension step of cycling, when the temperature used was 62°C instead of 64°C. The primers and probes for NoV-GI and -GII detection targeted the open reading frame 1 (ORF1)-ORF2 junction region^29^^)^ [forward primer COG1F: 5’-CGY TGG ATG CGN TTY CAT GA-3’, COG2F: 5’-CAR GAR BCN ATG TTY AGR TGG ATG AG-3’, reverse primer COG1R: 5’-CTT AGA CGC CAT CAT CAT TYA C-3’, COG2R: 5’-TCG ACG CCA TCT TCA TTC ACA-3’, probe COGP1b: Cy5-5’-(TAO) AGA TCG CGG TCT CCT GTC CA-3’-IB-RQ, COG2P: Cy3-5’-TGG GAG GGC GAT CGC AAT CT-3’-IB-RQ]. All RNA samples were run in duplicate with internal amplification control (IAC RNA, BioGX #750-0001, Birmingham, AL) [forward primer IC46F: 5’-GAC ATC GAT ATG GGT GCC G-3’, reverse primer IC194R: 5’-AAT ATT CGC GAG ACG ATG CAG-3’, probe IACP: TxR -5’-TCT CAT GCG TCT CCC TGG TGA ATG TG-IB RQ-3’].

### Statistical Analysis

Statistical analyses were performed using the open source software OpenEpi (Version 3.01)^30^^)^. One-tailed Chi Square testing determined the level of difference between positive rates among different categories of samples. For the spiking study, a two-sided *t* test with a 95% Confidence Interval (CI) was used to determine the level of difference between Ct values across the different categories of samples at each separate level of spiked virus. Statistical significance was defined as P < 0.05. A 50% endpoint Limit of Detection (LOD_50_) was defined as the amount of virus spiked into 10 L of agricultural water that resulted in positive RT-qPCR detection in 50% of replicates and calculated with the Reed-Muench method^31^^)^.

## Results

### Farm Water Sources and Surveillance Results

We utilized two types of filters, DEUF and MMS, to collect samples from agricultural water used for irrigation (**Fig. 1.**). A total of 310 samples were screened (nGA=143, nOH=167), consisting of 129 samples derived from DEUF filtration and 181 from MMS (**Table 1. and 2**.). Most samples collected (n = 241) were from surface waters (ponds, streams, creeks, and drip tapes); 69 samples were collected from ground water (wells). There were 117 samples collected from the end of the drip tapes, and 193 samples collected from other water sources.

**Table 1.** Summary of the prevalence of NoV and HAV in agricultural water on OH and GA farms during 2019 and 2020 growing seasons.

Sampling Year	CollectionMethod	Location	Month	Number of Samples Analyzed	Number of NoV-GI Positive Samples (%)	Number of NoV-GII Positive Samples (%)	Number of HAV Positive Samples (%)
2019	MMS	GA	May - Oct.	23	0 (0%)	0 (0%)	1 (4.3%)
		OH	June - Dec.	77	0 (0%)	3 (3.9%)	5 (6.5%)
2020	DEUF	GA	June - Sept.	80	0 (0%)	3 (3.8%)	12 (15%)
		OH	July - Sept.	49	0 (0%)	2 (4.1%)	8 (16%)
	MMS	GA	June - Sept.	40	0 (0%)	6 (15.0%)	2 (5.0%)
		OH	June - Sept.	41	1 (2.4%)	12 (29.3%)	1 (2.4%)
Total				310	1 (0.3%)	26 (8.4%)	29 (9.4%)

**Table 2.** Overall virus prevalence by collection method, state, water source, and month.

Classification	Catergory	Number of Samples Analyzed	Number of NoV-GII Positive Samples (%)	Number of HAV Positive Samples (%)
Subtotal by Collection Method	DEUF	129	5 (3.9%)	20 (15.5%)
	MMS	181	21 (11.6%)	9 (5.0%)
Subtotal by State	GA	143	9 (6.3%)	15 (10.5%)
	OH	167	17 (10.2%)	14 (8.4%)
Surface vs. Well	Surface	241	21 (8.7%)	22 (9.1%)
	Well	69	5 (7.2%)	7 (10.1%)
Drip Tape vs. Source	Drip Tape	117	11 (9.4%)	16 (13.7%)
	Source	193	15 (7.8%)	13 (6.7%)
Subtotal by Month	May	6	0 (0%)	0 (0%)
	June	57	10 (17.5%)	9 (15.8%)
	July	83	5 (6.0%)	6 (7.3%)
	August	78	6 (7.7%)	10 (12.8%)
	September	56	5 (8.9%)	4 (7.1%)
	October	22	0 (0%)	0 (0%)
	December	8	0 (0%)	0 (0%)

The water temperature during sampling at both GA and OH farms was between 15.6°C to 37.3°C. The eluates obtained from DEUF or MMS filtration differed significantly in color and turbidity, most likely due to the different nature of sources and locations. Prior to sampling the water from OH farms, the pH and oxidation-reduction potential (ORP) were recorded and found to range from pH 6.62 to 9.85 and ORP 18 to 250.

Viral surveillance data are summarized in Tables 1. and 2. NoV-GII was detected in 26 samples (5 DEUF, 21 MMS), which came from 21 sampling sites across 13 farms (OH and GA). The overall positive rate for NoV-GII was 8.4% with no statistically significant difference between samples from OH versus GA farms (p=0.110). The Ct values of NoV-GII ranged from 34.6 to 45.4. NoV-GII was detected in both surface and ground water with no significant difference between these two categories (p=0.349) nor between water taken from drip tapes versus other source water (p=0.308). NoV-GI was detected only in one pond sample from OH, which also contained NoV-GII.

HAV was detected in 29 samples (20 DEUF, 9 MMS), which came from 19 sampling sites on 11 farms across OH and GA. The overall positive rate for HAV was 9.4% with no significant difference between samples from OH versus GA farms (p=0.263). The Ct values ranged from 38.5 to 46.0. HAV was detected in both surface and ground water with no significant difference in prevalence across these source types (p=0.399). However, the positive rate for HAV was higher in drip tape samples than in other source water (p<0.05).

Over the surveillance period, 24 out of 36 (67%) of sampling sites were positive for one or more enteric viruses at least once during the months of June to September. We detected NoV-GII and HAV either simultaneously or in consecutive months on eight farms. However, detection results differed between the two filtration types: neither NoV nor HAV were detected at the same sampling site from DEUF and MMS at the same time. In most cases, the samples were only positive in one of the PCR duplicate reactions. A small percentage of positive samples could be confirmed by RT-qPCR after a further concentration of RNA by Amicon Ultra Centrifugal Filter 100 kDa MWCO (Sigma). The co-concentration of PCR inhibitors was observed in a small portion of samples, judged by a higher Ct value of Internal Amplification Control (IAC) in RT-qPCR. An additional step of RNA purification using Zymo OneStep PCR Inhibitor Removal kit could decrease the Ct values of the IAC, indicating the reduction of inhibitors (data not shown).

### Method Comparison Results: DEUF Versus MMS Filtration

Next, we evaluated the performance of the two filtration methods for virus recovery using artificially spiked stream water. To this end, we seeded 10L of water with three levels of NoV GII.6[P7] (ranging from 5.0E+05 to 5.7E+01 gc) using non-spiked water as a control, in triplicate. As determined by RT-qPCR results, the DEUF method consistently performed better than the MMS method (**Table 3****.**). In samples where viral RNA was detectable, the detection rate in samples derived from DEUF filtration was higher and the Ct value was lower than samples derived from MMS filtration at the same spiking level (p<0.05 at spiking level of 5.0E+05 gc, p=0.083 at spiking level of 2.2E+04 gc). The limit of detection (LOD_50_) was 4.9E+03 gc for the DEUF method and 1.1E+05 gc for the MMS method.

**Table 3.** Summary of detection results of pre-filtration NoV-spiked MMS and DEUF samples.

pre-Filtration NoV-GII.6 [P7] Spiking Level (gc/10 L)	MMS (with 0.45 µm filtration)		DEUF (with 0.45 µm filtration)		DEUF (without 0.45 µm filtration)	
	Detection Rate	Ct Value	Detection Rate	Ct Value	Detection Rate	Ct Value
5.0E+05	2/3	40.1 ± 1.0^**^	3/3	35.5 ± 0.2^**^	3/3	33.8 ± 0.3
2.2E+04	1/3	42.0 ± 0.1	3/3	39.0 ± 0.8	3/3	37.6 ± 1.1
1.3E+03	0/3	UD	0/3	UD	1/3	42.0^*^
5.7E+01	0/3	UD	0/3	UD	0/3	UD
0.0E+00	0/3	UD	0/3	UD	0/3	UD

gc, genome copy. Ct, cycle threshold. UD, undetectable.

* Only one of the RT-qPCR duplicate reactions in one of the three spiked samples was positive.

** The asterisk signifies a significant difference between the Ct values of MMS and DEUF samples at the pre-filtration spiking level of NoV-GII 5.0E+05 gc/10L (p<0.05).

### Comparison of Secondary Concentration Methods between PEG Precipitation and Ultracentrifugation

Given the large collection volumes of water, a two-step concentration of viral particles was necessary for both DEUF and MMS methods. To rule out the possibility that the difference we observed in virus recovery between these two methods derived from the specifically paired secondary concentration procedure, e.g., PEG precipitation for DEUF and ultracentrifugation for MMS, we carried out the spiking of NoV-GII.6[P7] at the post-elution step and compared the virus recovery between these two secondary concentration methods. The Ct values for samples recovered from PEG precipitation and ultracentrifugation in each method are shown in **Table 4****.** No statistically significant differences were found between these two secondary concentration methods (P = 0.142, 0.494, and 0.281 at the spiking level of 5.0E+05, 2.2E+04, and 1.3E+03 gc, respectively).

**Table 4.** Summary of detection results of post-elution NoV-spiked MMS and DEUF samples for comparison of ultracentrifugation and PEG precipitation.

post-Elution NoV-GII.6[P7] Spiking Level (gc/sample)	Ultracentrifugation for MMS		PEG ppt. for DEUF	
	Detection Rate	Ct Value	Detection Rate	Ct Value
5.0E+05	3/3	32.7 ± 0.8	3/3	34.4 ± 1.4
2.2E+04	3/3	37.4 ± 0.8	3/3	38.1 ± 1.4
1.3E+03	2/3	41.9 ± 1.3	3/3	40.5 ± 1.1
5.7E+01	0/3	UD	0/3	UD
0.0E+00	0/3	UD	0/3	UD

ppt., precipitation.

### Effect of Bacteria Removal on Viral Recovery

Agricultural water usually comes in variable turbidity with suspended solids, inorganic materials, algae, organic matters, and microorganisms including bacteria. Most of these materials will be captured by the filters and co-eluted with viruses during the processing. To clarify the eluate, we used high-speed centrifugation (10,000 x g) and subsequently 0.45µm membrane filtration to remove most of the solids and bacteria. However, when we skipped the step of 0.45µm membrane filtration in DEUF method, we observed an improvement in viral recovery (**Table 3.**). This lowering of Ct values was significant at the spiking level of 5.5E+05 gc (p<0.05), although not significant at the spiking level of 2.2E+04 gc (p=0.149).

## Discussion

Foodborne illnesses can be transmitted through food products contaminated with viral pathogens. Key domestic products vulnerable to contamination include leafy greens, soft berries, and bivalve shellfish. Forty-six percent of foodborne illnesses were attributed to produce^32^^)^. In recent years, several hepatitis A outbreaks in the U.S. have been traced to consumption of fresh and frozen produce imported from regions where HAV is endemic or where hygiene compliance and food safety standards enforcement are inadequate^33^^)^. Although no enteric virus outbreaks linked to domestically grown berries have been reported in 35 years, outbreaks associated with imported fresh and frozen berries have occurred^34^^)^. A recent example is the 2023 multistate outbreak of hepatitis A virus infections linked to frozen organic strawberries imported from Mexico^35^^)^. Although viral pathogens contaminating fresh produce might originate from workers infected with these pathogens, environmental water is increasingly considered a potential source or reservoir for the pathogens^11^^)^. Reliable microbiological analyses of agricultural water are fundamental to food safety, as any sewage (onsite or distal) polluting water sources used for irrigation or processing could contaminate fresh produce. Therefore, improving methods for detecting viral pathogens to help better control foodborne outbreaks are imperative. Current methods for concentration, isolation, extraction, and detection methods have technical limitations which cannot be overcome by culturing methods, as most viruses are difficult to propagate *in vitro*. This means efficient extraction and sensitive molecular detection methods are vital for meaningful monitoring of water quality.

In this surveillance study, we attempted to address some of the challenges in assessing the level or presence/absence of human viral pathogens in irrigation water, including the need to concentrate any virus present to enhance detection and fluctuations in virus levels over time. Enteric viruses are likely to be present only in low concentrations and as transient presences in the environment. There are likely seasonal fluctuations in contamination levels, influenced by the likelihood of disease outbreaks in the local population, or inappropriate sanitation practices on/near the farms. Furthermore, whether any viral pathogens in a moving body of water enter an agricultural water system is a function of not only disease incidence but also sewage runoffs, each of which is constantly changing over time, given the dynamic nature of rivers and streams. The physicochemical and microbiological composition of water sources change constantly, inevitably affecting the homogeneity of sampling and performance of detection assays.

Our investigation sampled traditional irrigation sources, including creeks, ponds, streams, and wells at 36 different sites representing 21 farms in two states over the course of 2 years. We found the prevalence of HAV or NoV in these GA and OH water sources to be comparable to the ranges reported by other studies^9^^)^. Interestingly, although HAV is as prevalent in the environment as NoV, it has caused fewer cases of foodborne illnesses and outbreaks, according to the Centers for Disease Control and Prevention (CDC) case reports^36^^)^. One reason could be the availability of a vaccine for HAV; there is no vaccine for NoV. Scientific evidence indicates that HAV has a notably slow rate of molecular evolution and high genetic stability compared to the rapid evolution and high mutation rates observed in noroviruses^37^^,^^38^^,^^39^^,^^40^^,^^41^^)^. HAV’s genetic stability has enabled successful vaccine development with long-lasting immunity, while NoV’s rapid evolution has complicated vaccine development efforts and contributes to the lack of long-lasting cross-protective immunity following natural infection. Hepatitis A vaccine, introduced in 1995 in the U.S., led to a 95% decrease in reported cases from 1996 to 2011^42^^)^. According to a 2011 study, an estimated 1,566 foodborne HAV infections occur in the U.S. every year^43^^)^. In 2016, national vaccination coverage for 1 and ≥2 doses of hepatitis A vaccine among adolescents (13-17 years) was 73.9% and 64.4%, respectively^44^^)^. However, in 2021, self-reported vaccination data showed that only 24.8% of adults aged 19 and older had received at least one dose of hepatitis A vaccine^42^^,^^45^^)^. Widespread outbreaks re-emerged in 2016 among adults, highlighting the need for sustained, high vaccination coverage^46^^)^.

Although other studies have found norovirus levels in the environment to be highly concentrated during the winter months, our work focused on the growing seasons thus did not directly address the effects of seasonality. The relatively higher prevalence of viruses we observed from June to September than those in May or October or December is more likely to be attributed to our sampling size and the higher level of human activity on these farms. Of the predominant norovirus genotypes that infect humans (i.e., NoV-GI and -GII), NoV-GII had been observed at higher densities in wastewater than NoV-GI^14^^)^. This is consistent with our finding that NoV-GII was more dominant than NoV-GI in agricultural water.

As the levels of foodborne viruses are expected to be low in agricultural water, the first step for monitoring viruses is to perform primary concentration from large volumes of water. Hence, we assessed the analytical performance of both DEUF and MMS to concentrate viruses from 10 L of water. Both of these two “trapping” methods offer large surface areas, require no preconditioning of water samples, e.g. pH adjustment^47^^)^, thus tolerate water with high turbidity and enable on-site sampling of large volumes. While DEUF filters are commercially available, as of mid-2024 MMS are not, which creates an in-house requirement for making these swabs - this is both time-consuming and difficult to standardize. Our results from the spiking study demonstrate DEUF performed better for virus recovery than MMS, judged by the higher detection rates and lower Ct values from the same spiking levels of NoV-GII in DEUF samples. This conclusion was confirmed after we verified that the two secondary concentration methods, PEG precipitation and ultracentrifugation, are comparably efficient for recovering viruses from samples. PEG precipitation is a classic method often used in International Organization for Standardization (ISO) protocols^48^^,^^49^^)^. It can process large volumes of water, providing an easy and convenient method to concentrate viruses and remove impurities without expensive ultracentrifugation equipment. Our study suggests PEG precipitation provides an effective, low-cost procedure with high sample throughput that can be performed in a minimally equipped laboratory. It is noted that, in 2020 surveillance, the MMS method appeared to detect more NoV-GII and less HAV than the DEUF method (Table 1**.**). However, this surveillance study was not designed for such method comparison. The samples might be collected from different sites at different time periods. Our spiked experiments confirmed the improved recovery of NoV-GII using the DEUF method. It might be worthwhile to perform a similar method comparison study for HAV in the future.

Our data indicated that viral recovery rates decreased when the eluate went through an extra step of membrane filtration (Table 3.). Studies have shown that viruses in water frequently become attached to all sizes of suspended organic and inorganic matters, and even adhere to bacteria^50^^)^. Therefore, the removal of solids after high-speed centrifugation and subsequent filtration of eluates using a 0.45 µm membrane was highly likely to affect viral recovery. Such clarification may cause the loss of particle-associated viruses before PEG precipitation. Whether or not to include clarification steps depends on the selection of downstream analysis methods. For RT-qPCR, the presence of bacterial nucleic acids might not affect the specific amplification of viral targets. However, for whole genome sequencing (WGS) and cell culture assays, removing those bacteria will be preferable: in a WGS analysis of an MMS-derived sample, using 0.45 µm membrane filtration removed most of the bacteria, resulting in a cleaner sequencing background for virus detection compared to non-filtered samples (data not shown).

The relatively high LOD_50_ (10^3^ – 10^5^ gc/10 L) and high Ct values (close to or greater than 40) suggest the need for method improvement in order to increase the sensitivity for the low viral load samples. This study utilized only the RT-qPCR protocols specified in FDA BAM Chapter 26. Alternative primer and probe sets described in other documents, such as ISO 15216, were not evaluated. Ideally, the PCR results should be confirmed by alternative methods such as WGS^51^^)^ or cell culture if possible. Identifying human fecal indicators, such as CrAssphage or pepper mild mottle virus (PMMoV), in agricultural water, would be another way to determine whether there is contamination and thus the possible presence of human pathogens^52^^,^^53^^,^^54^^,^^55^^)^. While CrAssphage is a bacteriophage highly specific to human gut *Bacteroides* species, PMMoV is more of a diet-derived marker from pepper consumption. PMMoV can vary significantly based on population dietary habits and geographic location. Moreover, these indicators do not necessarily correlate well with viral contamination. Although viruses and these fecal indicators may be present together in sewage, their environmental fate differs once released. Dilution effects and variations in stability and persistence can cause viruses and indicators to disperse along separate pathways. Nevertheless, these indicators could play an important role in assessing agricultural water quality. Molecular based methods such as RT-qPCR cannot discriminate between infectious and non-infectious particles, or naked RNA. It is possible for inactivated HAV RNA to be detected in water for as long as three months after inoculation and storage^56^^)^. While the presence of non-infectious viral RNA in a water source may confound the investigations of current outbreaks, that presence can indicate the likelihood that somewhere in the waterway there has been contamination by human waste.

Our results address two critical data gaps, namely, foodborne virus prevalence in surface and ground waters in the two regions of the United States, and subsequent method evaluation of primary and secondary concentration of viruses from large volumes of water. It is important to protect water sources from human feces to avoid potential food contamination. The approaches and methodologies used in this study will provide suitable options to investigate the prevalence of foodborne viruses in agricultural water sources, identify potential contamination routes, and conduct root cause analyses.

## References

[1] EFSA Panel on Biological Hazards (BIOHAZ), Koutsoumanis K, Ordóñez AA, Bolton D, et al.. Microbiological hazards associated with the use of water in the post‐harvest handling and processing operations of fresh and frozen fruits, vegetables and herbs (ffFVHs). Part 1 (outbreak data analysis, literature review and stakeholder questionnaire). EFSA J. 2023; 21(11): e08332. 10.2903/j.efsa.2023.8332 37928944 PMC10623241

[2] Hirneisen KA, Kniel KE. Comparative uptake of enteric viruses into spinach and green onions. Food Environ Virol. 2013; 5(1): 24–34. 10.1007/s12560-012-9093-x 23412715

[3] Hirneisen KA, Sharma M, Kniel KE. Human enteric pathogen internalization by root uptake into food crops. Foodborne Pathog Dis. 2012; 9(5): 396–405. . 10.1089/fpd.2011.1044 22458717

[4] Yang Z, Chambers H, DiCaprio E, Gao G, Li J. Internalization and dissemination of human norovirus and Tulane virus in fresh produce is plant dependent. Food Microbiol. 2018; 69: 25–32. 10.1016/j.fm.2017.07.015 28941906 PMC6361382

[5] Esseili MA, Meulia T, Saif LJ, Wang Q. Tissue distribution and visualization of internalized human norovirus in leafy greens. Appl Environ Microbiol. 2018; 84(12): e00292-18. 10.1128/AEM.00292-18 29625983 PMC5981073

[r6] 6.Callejón RM, Rodríguez-Naranjo MI, Ubeda C, Hornedo-Ortega R, Garcia-Parrilla MC, Troncoso AM. Reported foodborne outbreaks due to fresh produce in the United States and European Union: Trends and causes. Foodborne Pathog Dis.., 2015;12 (1):32-38.. Published January 1,2015 10.1089/fpd.2014.1821 25587926

[7] Chatziprodromidou IP, Bellou M, Vantarakis G, Vantarakis A. Viral outbreaks linked to fresh produce consumption: a systematic review. J Appl Microbiol. 2018; 124(4): 932–942. 10.1111/jam.13747 29485236

[8] Rodríguez RA, Gundy PM, Rijal GK, Gerba CP. The impact of combined sewage overflows on the viral contamination of receiving waters. Food Environ Virol. 2012; 4(1): 34–40. 10.1007/s12560-011-9076-3 23412766

[9] Boehm AB, Silverman AI, Schriewer A, Goodwin K. Systematic review and meta-analysis of decay rates of waterborne mammalian viruses and coliphages in surface waters. Water Res. 2019; 164: 114898. 10.1016/j.watres.2019.114898 31404902

[10] Anderson-Coughlin BL, Craighead S, Kelly A, et al. Enteric viruses and pepper mild mottle virus show significant correlation in select Mid-Atlantic agricultural waters. Appl Environ Microbiol. 2021; 87(13): e00211-21. 10.1128/AEM.00211-21 33893119 PMC8316098

[11] Tian P, Yang D, Shan L, et al. Concurrent detection of human norovirus and bacterial pathogens in water samples from an agricultural region in Central California Coast. Front Microbiol. 2017; 8: 1560. 10.3389/fmicb.2017.01560 28871242 PMC5566579

[r12] 12.Federal Provincial Territorial Committee on Drinking Water. *Guidelines for Canadian drinking water quality: Guideline technical document – Enteric Viruses* ; 2019.

[13] Montazeri N, Goettert D, Achberger EC, Johnson CN, Prinyawiwatkul W, Janes ME. Pathogenic enteric viruses and microbial indicators during secondary treatment of municipal wastewater. Appl Environ Microbiol. 2015; 81(18): 6436–6445. 10.1128/AEM.01218-15 26162869 PMC4542245

[14] Eftim SE, Hong T, Soller J, et al. Occurrence of norovirus in raw sewage – A systematic literature review and meta-analysis. Water Res. 2017; 111: 366–374. 10.1016/j.watres.2017.01.017 28110140

[15] Teunis PF, Moe CL, Liu P, et al. Norwalk virus: how infectious is it? J Med Virol. 2008; 80(8): 1468–1476. 10.1002/jmv.21237 18551613

[16] Yezli S, Otter JA. Minimum infective dose of the major human respiratory and enteric viruses transmitted through food and the environment. Food Environ Virol. 2011; 3(1): 1–30. 10.1007/s12560-011-9056-7 35255645 PMC7090536

[17] Cuevas-Ferrando E, Randazzo W, Pérez-Cataluña A, Sánchez G. HEV occurrence in waste and drinking water treatment plants. Front Microbiol. 2020; 10: 2937. 10.3389/fmicb.2019.02937 31993027 PMC6971180

[18] Gerrity D, Papp K, Stoker M, Sims A, Frehner W. Early-pandemic wastewater surveillance of SARS-CoV-2 in Southern Nevada: Methodology, occurrence, and incidence/prevalence considerations. Water Res X. 2021; 10: 100086. 10.1016/j.wroa.2020.100086 33398255 PMC7774458

[19] Liu P, Ibaraki M, Kapoor R, et al. Development of Moore swab and ultrafiltration concentration and detection methods for *Salmonella* Typhi and *Salmonella* Paratyphi A in wastewater and application in Kolkata, India and Dhaka, Bangladesh. Front Microbiol. 2021; 12: 684094. 10.3389/fmicb.2021.684094 34335510 PMC8320291

[20] Kahler AM, Mattioli MC, da Silva AJ, Hill V. Detection of *Cyclospora cayetanensis* in produce irrigation and wash water using large-volume sampling techniques. Food Waterborne Parasitol. 2021; 22: e00110. 10.1016/j.fawpar.2021.e00110 33681488 PMC7930117

[21] Forés E, Rusiñol M, Itarte M, Martínez-Puchol S, Calvo M, Bofill-Mas S. Evaluation of a virus concentration method based on ultrafiltration and wet foam elution for studying viruses from large-volume water samples. Sci Total Environ. 2022; 829: 154431. 10.1016/j.scitotenv.2022.154431 35278558

[22] Yang Z, Mammel M. Near-complete genome sequence of a human norovirus GII.P7-GII.6 strain detected in a Maryland patient in 2018. Microbiol Resour Announc. 2019; 8(16): e00191-19. 10.1128/MRA.00191-19 31000549 PMC6473143

[23] Sbodio A, Maeda S, Lopez-Velasco G, Suslow TV. Modified Moore swab optimization and validation in capturing E. coli O157:H7 and *Salmonella enterica* in large volume field samples of irrigation water. Food Res Int. 2013; 51(2): 654–662. . 10.1016/j.foodres.2013.01.011

[r24] 24.Durigan M, Murphy H, Deng K, et al. FDA BAM 19c: Dead-end ultrafiltration for the detection of Cyclospora cayetanensis from agricultural water. 2020. 10.1128/AEM.01595-20 PMC7657621 32948525

[25] Cuevas-Ferrando E, Allende A, Pérez-Cataluña A, et al. Occurrence and accumulation of human enteric viruses and fhages in process water from the fresh produce industry. Foods. 2021; 10(8): 1853. 10.3390/foods10081853 34441630 PMC8391481

[26] Liu P, Hill VR, Hahn D, et al. Hollow-fiber ultrafiltration for simultaneous recovery of viruses, bacteria and parasites from reclaimed water. J Microbiol Methods. 2012; 88(1): 155–161. 10.1016/j.mimet.2011.11.007 22108496

[27] Williams-Woods J, Rodriguez R, Marchant J, Swinford AG, Burkhardt W III 2022.

[28] Gardner SN, Kuczmarski TA, Vitalis EA, Slezak TR. Limitations of TaqMan PCR for detecting divergent viral pathogens illustrated by hepatitis A, B, C, and E viruses and human immunodeficiency virus. J Clin Microbiol. 2003; 41(6): 2417–2427. 10.1128/JCM.41.6.2417-2427.2003 12791858 PMC156483

[29] Kageyama T, Kojima S, Shinohara M, et al. Broadly reactive and highly sensitive assay for Norwalk-like viruses based on real-time quantitative reverse transcription-PCR. J Clin Microbiol. 2003; 41(4): 1548–1557. 10.1128/JCM.41.4.1548-1557.2003 12682144 PMC153860

[30] OpenEpi. Open Source Epidemiologic Statistics for Public Health. http://www.openepi.com/Menu/OE_Menu.htm.

[31] Lei C, Yang J, Hu J, Sun X. On the calculation of TCID_50_ for quantitation of virus infectivity. Virol Sin. 2021; 36(1): 141–144. 10.1007/s12250-020-00230-5 32458296 PMC7973348

[32] Painter JA, Hoekstra RM, Ayers T, et al. Attribution of foodborne illnesses, hospitalizations, and deaths to food commodities by using outbreak data, United States, 1998-2008. Emerg Infect Dis. 2013; 19(3): 407–415. 10.3201/eid1903.111866 23622497 PMC3647642

[33] Hu X, Collier MG, Xu F. Hepatitis A outbreaks in developed countries: Detection, control, and prevention. Foodborne Pathog Dis. 2020; 17(3): 166–171. 10.1089/fpd.2019.2648 31829731

[r34] 34.U.S. Food and Drug Administration. Summary of FDA’s strategy to prevent human norovirus and hepatitis A outbreaks associated with fresh and frozen berries. 2025.

[r35] 35.U.S. Centers for Disease Control and Prevention. Hepatitis A outbreak linked to frozen organic strawberries. 2024.

[36] U.S. Centers for Disease Control and Prevention. Surveillance for foodborne disease outbreaks, United States 2017 Annual Report. Atlanta, Georgia. U.S. Department of Health and Human Services. 2019.

[37] Moratorio G, Costa-Mattioli M, Piovani R, Romero H, Musto H, Cristina J. Bayesian coalescent inference of hepatitis A virus populations: evolutionary rates and patterns. J Gen Virol. 2007; 88(11): 3039–3042. 10.1099/vir.0.83038-0 17947528

[38] Kulkarni MA, Walimbe AM, Cherian S, Arankalle VA. Full length genomes of genotype IIIA hepatitis A virus strains (1995–2008) from India and estimates of the evolutionary rates and ages. Infect Genet Evol. 2009; 9(6): 1287–1294. 10.1016/j.meegid.2009.08.009 19723592

[39] Vaughan G, Goncalves Rossi LM, Forbi JC, et al. Hepatitis A virus: Host interactions, molecular epidemiology and evolution. Infect Genet Evol. 2014; 21: 227–243. 10.1016/j.meegid.2013.10.023 24200587

[40] Majeed AA, Sarfraz M, Butt AS. Evolving trends in hepatitis A epidemiology: Shifting patterns, emerging risks, and future strategies. World J Virol. 2025; 14(4): 112590. 10.5501/wjv.v14.i4.112590 41479578 PMC12754557

[41] Omatola CA, Mshelbwala PP, Okolo MLO, et al. Noroviruses: Evolutionary dynamics, epidemiology, pathogenesis, and vaccine advances—a comprehensive review. Vaccines (Basel). 2024; 12(6): 590. 10.3390/vaccines12060590 38932319 PMC11209302

[42] Foster MA, Haber P, Nelson NP. Chapter 9: Hepatitis A/Pink Book - CDC. 2024.

[43] Scallan E, Hoekstra RM, Angulo FJ, et al. Foodborne illness acquired in the United States--major pathogens. Emerg Infect Dis. 2011; 17(1): 7–15. 10.3201/eid1701.P11101 21192848 PMC3375761

[44] Nelson NP, Yankey D, Singleton JA, Elam-Evans LD. Hepatitis A vaccination coverage among adolescents (13–17 years) in the United States, 2008–2016. Vaccine. 2018; 36(12): 1650–1659. 10.1016/j.vaccine.2018.01.090 29449100 PMC5895091

[r45] 45.Hung MC, Srivastav A, Lu PJ, et al. Vaccination coverage among adults in the United States, national health interview survey, 2021. U.S. Centers for Disease Control and Prevention AdultVaxView. 2024.

[46] Nelson NP, Weng MK, Hofmeister MG, et al. Prevention of hepatitis A virus infection in the United States: Recommendations of the advisory committee on immunization practices, 2020. MMWR Recomm Rep. 2020; 69(5): 1–38. 10.15585/mmwr.rr6905a1 32614811 PMC8631741

[47] Cashdollar JL, Wymer L. Methods for primary concentration of viruses from water samples: a review and meta-analysis of recent studies. J Appl Microbiol. 2013; 115(1): 1–11. 10.1111/jam.12143 23360578

[r48] 48.International Organization for Standardization. ISO/TC34/SC9 15216–1:2017. Microbiology of the food chain - Horizontal method for determination of hepatitis A virus and norovirus using real-time RT-PCR - Part 1: Method for quantification. 2017.

[49] Lowther JA, Bosch A, Butot S, et al. Validation of EN ISO method 15216 - Part 1 – Quantification of hepatitis A virus and norovirus in food matrices. Int J Food Microbiol. 2019; 288: 82–90. 10.1016/j.ijfoodmicro.2017.11.014 29229293

[50] Wu H, Brighton K, Aw TG. Quantification and characterization of particle-associated viruses in secondary effluent wastewater for water reuse. presented at: American Society for Microbiology Microbe annual conference; June 15-19, 2023 2023; Houston, Texas.

[51] Yang Z, Kulka M, Yang Q, et al. Whole-Genome sequencing-based confirmatory methods on RT-qPCR results for the detection of foodborne viruses in frozen berries. Food Environ Virol. 2024; 16(2): 225–240. 10.1007/s12560-024-09591-6 38687458 PMC11186866

[52] Park GW, Ng TFF, Freeland AL, et al. CrAssphage as a novel tool to detect human fecal contamination on environmental surfaces and hands. Emerg Infect Dis. 2020; 26(8): 1731–1739. 10.3201/eid2608.200346 32511090 PMC7392416

[53] Wu H, Juel MAI, Eytcheson S, Aw TG, Munir M, Molina M. Temporal and spatial relationships of CrAssphage and enteric viral and bacterial pathogens in wastewater in North Carolina. Water Res. 2023; 239: 120008. 10.1016/j.watres.2023.120008 37192571 PMC10896230

[54] Suh SH, Lee JS, Kim SH, Vinjé J, Kim SH, Park GW. Evaluation of crAssphages as a potential marker of human viral contamination in environmental water and fresh leafy greens. Front Microbiol. 2024; 15: 1374568. 10.3389/fmicb.2024.1374568 38618485 PMC11010641

[r55] 55.Kitajima M, Sassi HP, Torrey JR. Pepper mild mottle virus as a water quality indicator. *npj Clean Water*. 2018;1 (19). . 10.1038/s41545-018-0019-5

[56] Trudel-Ferland M, Jubinville E, Jean J. Persistence of hepatitis A virus RNA in water, on non-porous surfaces, and on blueberries. Front Microbiol. 2021; 12: 618352. 10.3389/fmicb.2021.618352 33613487 PMC7890088

